# Interaction of Ampicillin and Amoxicillin with Mn^2+^: A Speciation Study in Aqueous Solution

**DOI:** 10.3390/molecules25143110

**Published:** 2020-07-08

**Authors:** Claudia Foti, Ottavia Giuffrè

**Affiliations:** Dipartimento di Scienze Chimiche, Biologiche, Farmaceutiche ed Ambientali, Università di Messina, Viale F. Stagno d’Alcontres 31, 98166 Messina, Italy; cfoti@unime.it

**Keywords:** ampicillin, amoxicillin, Mn(II), speciation profile in aqueous solution, sequestration, potentiometric, spectrophotometric titrations

## Abstract

A potentiometric and UV spectrophotometric investigation on Mn^2+^-ampicillin and Mn^2+^-amoxicillin systems in NaCl aqueous solution is reported. The potentiometric measurements were carried out under different conditions of temperature (15 ≤ *t*/°C ≤ 37). The obtained speciation pattern includes two species for both the investigated systems. More in detail, for system containing ampicillin MLH and ML species, for that containing amoxicillin, MLH_2_ and MLH ones. The spectrophotometric findings have fully confirmed the results obtained by potentiometry for both the systems, in terms of speciation models as well as the stability constants of the formed species. Enthalpy change values were calculated via the dependence of formation constants of the species on temperature. The sequestering ability of ampicillin and amoxicillin towards Mn^2+^ was also evaluated under different conditions of pH and temperature via pL_0.5_ empirical parameter (i.e., cologarithm of the ligand concentration required to sequester 50% of the metal ion present in traces).

## 1. Introduction

Photosynthesis, proteoglycan biosynthesis and antioxidative actions are the main biochemical functions [1]. Mn^2+^ is the most stable thermodynamically oxidation state in both acidic and basic solutions [2,3,4]. In biological systems Mn^2+^ can be substituted and can substitute other divalent cations, such as Ca^2+^, Mg^2+^, Fe^2+^. Mn^2+^ and Mg^2+^ may exchange cofactor roles in enzyme reactions and they show the same affinity for simple oxygen donors, such as polyphosphates [5,6]. The main difference among Mn(II) and Mg(II) chemistry consists in the higher affinity of manganese ion for nitrogen or sulphur ligands. It presents an intermediate size and an intermediate electron acceptor strength between Ca(II) and Mg(II). In biological systems, Mn(II) concentration is about 0.1 μmol L^−1^ inside and outside cells [6]. High manganese concentrations have been detected in the pancreas, liver, kidney and intestines, where the manganese content was found in the range 2.5–5.3 and 4.9–6.0 μg kg^−1^, in kidney and liver respectively [1]. In blood, manganese is bound to plasma proteins, its concentration in the whole blood is about 11 μg L^−1^ and in the serum 0.6–1.3 μg L^−1^. In the urine the mean concentration of 9.32 μg L^−1^ was found [7]. For humans the main sources of manganese are foods and beverages and its absorption occurs mainly through respiratory and gastrointestinal tracts [1]. In humans, animals and plants, manganese is an activator as well as a constituent of various enzymes [5]. As a consequence of its excess or deficiency numerous pathologies can occur [8]. Thereby, it acts as both an essential nutrient and toxic element. Under normal conditions, the body protects itself through the homeostatic mechanism, acting on absorption and excretion [1]. In humans, symptoms of both manganese deficiency and oral parenteral poisoning are rare [9]. Manganese is also widely present in waters, soils, sediments, air. Its concentration in the open oceans, mainly as Mn^2+^, range between 0.01 and 0.16 μg kg^−1^. More in detail, manganese concentration is in the range 1–500 μg L^−1^ in surface waters, 5–25 μg L^−1^ in drinking water [1]. The simple [Mn(H_2_O)_6_]^2+^ ion is commonly found in freshwaters and in seawaters [4]. Similarly to the other bivalent cations of the transition series, Mn^2+^ can be considered resistant to hydrolysis [2]. At ordinary concentrations, hydrolysis starts to be significant above pH 8 [2]. The interactions of Mn^2+^ with antibiotics are of fundamental importance both from biological and environmental point of view. The biological interest is more obvious than the environmental one, however, one must consider that antibiotics are excreted via feces and urine, through domestic and hospital wastewaters. They are thus released into aquatic environments, soils, sediments and many of them persist for long time in the environment [10]. In this context, it is crucial the study of their interactions with metal cations, in order to evaluate the mechanisms by which they can be retained in natural waters.

Among antibiotics, the aminopenicillins are semi-synthetic penicillins that can be considered as *N*-acylderivatives of 6-aminopenicillanic acid. Semi-synthetic β-lactam antibiotics have been designed for a wider spectrum of action and better pharmacokinetic properties than natural penicillin. Among semi-synthetic penicillins, ampicillin and amoxicillin have the best oral absorption and they distribute well in body fluids. They are commonly employed as antibiotics owing to the wide spectra mechanisms of action, since they stop bacteria proliferation [11]. It is known that the bioavailability of β-lactam antibiotics is influenced by their interaction in vivo with several metal cations [12,13,14,15,16,17]. For this reason, the assessment of the interactions of these antibiotics with metal cations which play biological roles in the human body is crucial [18]. Complexing abilities of the most important amino-penicillins, i.e., ampicillin and amoxicillin, with some metal cations, namely Zn^2+^, Cu^2+^, Ca^2+^ and Mg^2+^, were already studied [19,20,21,22].

**Figure 1:**
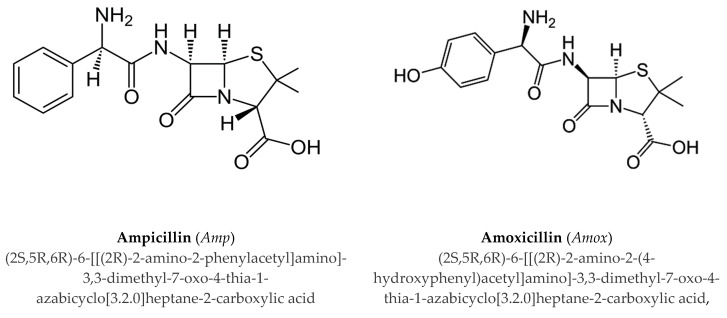
Ligands under study.

In this paper the interactions of Mn^2+^ with ampicillin (*Amp*) and amoxicillin (*Amox*), shown in Figure 1, were investigated. With the aim to clarify the in vivo mode of action of these antibiotics with a bioavailable metal cation, the study of the interactions of *Amp* and *Amox* with Mn^2+^ was performed [23]. The investigation on these systems was carried out by potentiometry and UV spectrophotometry. Potentiometric titrations were performed under different conditions of ionic strengths and temperature in order to model the dependence of formation constants on temperature and on ionic strength. Spectrophotometric titrations were used to confirm the speciation models and the stability of complex species.

Sequestering ability of *Amp* and *Amox* was quantified via a Boltzmann type equation where the sum of molar fractions of all Mn^2+^-ligand complex species were fitted vs. cologarithm of total ligand concentration. Sequestering ability under different conditions of temperature, pH and ionic strength was also evaluated.

## 2. Results and Discussion

### 2.1. Aqueous Behaviour and Speciation of Mn^2+^-Amp and Mn^2+^-Amox Species

In this investigation, potentiometric and UV spectrophotometric titrations were performed in order to define the best speciation models for both the investigated systems and to obtain reliable formation constants values of the complex species. Formation equilibria of metal cation(M)-ligand(L) species are the following (were the charges are omitted for simplicity):

M + L + rH = MLH_r_      β_r_ (1)

M + LH_r_ = MLH_r_       *K_r_* (2)

Hydrolysis constants of Mn^2+^ and protonation constants of *Amp* and *Amox*, were taken into account in the calculations (Tables S1 and S2, respectively of Supplemetary Materials). The elaboration of the potentiometric experimental data has allowed to obtain the results listed in Table 1. The choice of the best speciation model is always a very delicate phase in the study of the equilibria in solution. It was chosen on the basis of a series of fundamental criteria, such as the best statistical fit, the variance ratio between the accepted model and the others, the model simplicity, the formation percentages of the complex species [24,25]. Taking into account what has just been described, the speciation model chosen for the Mn^2+^(M)-*Amp*(L) system, under all the temperature and ionic strength conditions investigated, was found to contain only 1:1 M:L species, namely MLH and ML.

**Figure 2:**
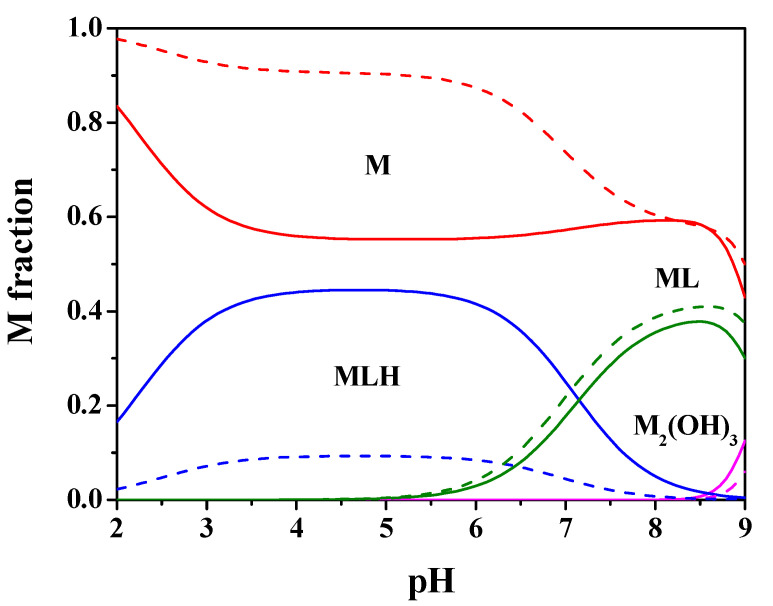
Speciation diagram of Mn^2+^-*Amp*(L) system, at C_M_ = 2 mmol L^−1^, C_L_ = 4 mmol L^−1^, *t* = 25 °C, *I* = 0.15 mol L^−1^ (solid line), *I* = 1 mol L^−1^ (dashed line).

Figure 2 shows the speciation diagram for this system at *t* = 25 °C, C_M_ = 2 mmol L^−1^, C_L_ = 4 mmol L^−1^, *I* = 0.15 mol L^−1^ in NaCl. It can be observed from the diagram that the MLH species is present in a wide pH range with metal fraction greater than 0.4, the ML one reaches almost 0.4 as a fraction at pH = 8.5. The M_2_(OH)_3_ hydrolytic species is significant only starting from pH > 9.

**Figure 3:**
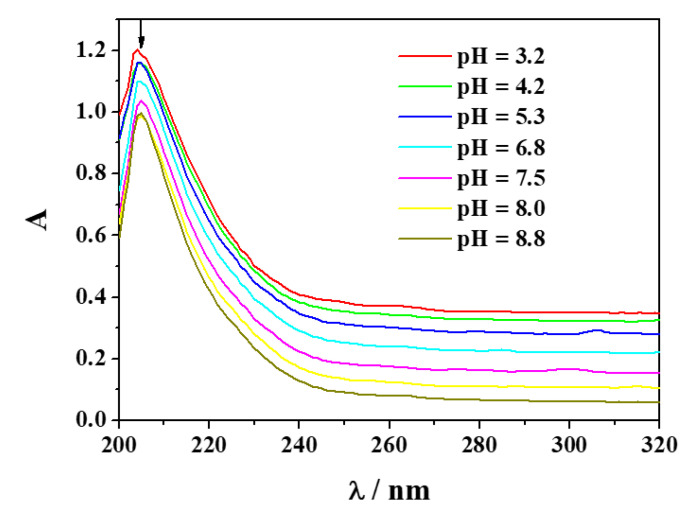
Experimental spectra at different pH on solutions containing Mn^2+^(M) and *Amp*(L) at C_M_ = 0.05 mmol L^−1^ and C_L_ = 0.05 mmol L^−1^, *t* = 25 °C, *I* = 0.15 mol L^−1^ in NaCl.

In order to confirm the speciation model and the formation constants gained via potentiometry by spectroscopic techniques as already established for other systems [26,27,28,29,30,31,32,33,34], spectrophotometric titrations on solutions containing Mn^2+^ and *Amp* at *t* = 25 °C and *I* = 0.15 mol L^−1^ were performed under different concentration conditions. As an example, experimental spectra recorded on solutions containing Mn^2+^ and *Amp* at C_M_ = 0.05 mmol L^−1^ and C_L_ = 0.05 mmol L^−1^, were depicted in Figure 3. It shows that the absorption spectra have a single maximum with a slight hypochromic and hypsochromic effect with increasing pH.

**Figure 4:**
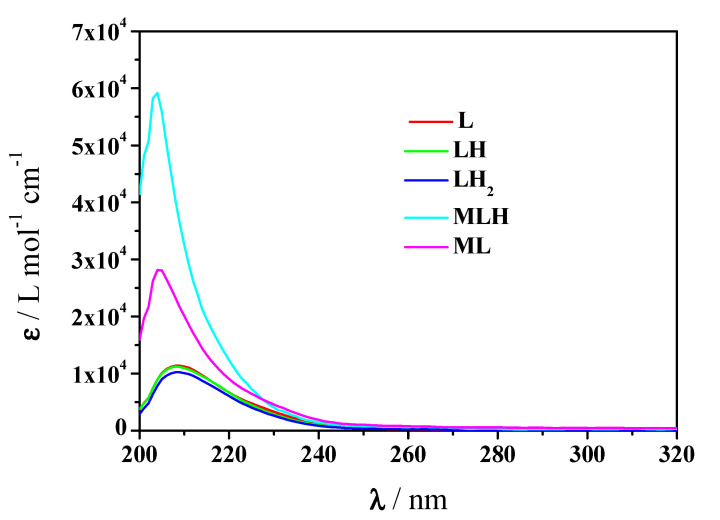
Molar absorbance values of protonated and unprotonated *Amp* species and Mn^2+^-*Amp* complexes at *t* = 25 °C and *I* = 0.15 mol L^−1^ in NaCl (charges omitted for simplicity).

Via the processing of the spectrophotometric experimental data with the HypSpec program the results listed in Table 2 were obtained, together with the potentiometric ones under the same conditions of temperature and ionic strength. An examination of both results shows that the speciation model proposed by potentiometry was confirmed by spectrophotometry and the formation constants of both complex species were obtained with very close values to those gained by potentiometry. The molar absorbances of *Amp* species and of Mn^2+^-*Amp* complexes at *t* = 25 °C and *I* = 0.15 mol L^−1^ in NaCl are represented in Figure 4. It is observed that both MLH and ML complex species have molar absorbances higher than those of the single L, LH, LH_2_ species of *Amp*.

**Figure 5:**
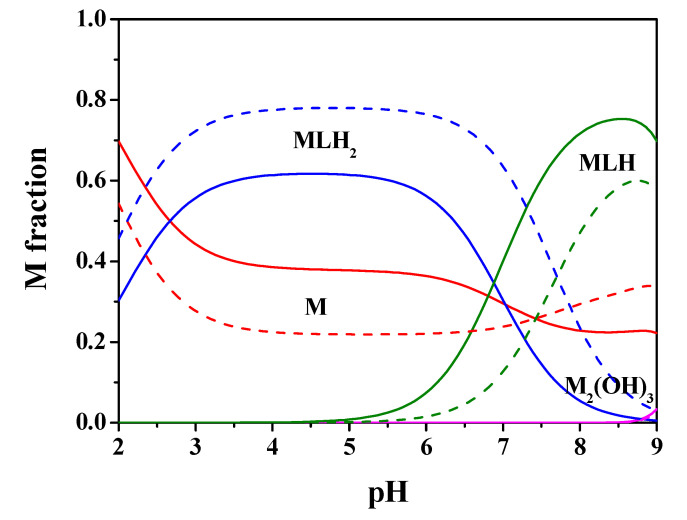
Speciation diagram of Mn^2+^-*Amox*(L) system, at C_M_ = 2 mmol L^−1^, C_L_ = 4 mmol L^−1^, *t* = 25 °C, *I* = 0.15 mol L^−1^ (solid line), *I* = 1 mol L^−1^ (dashed line).

The processing of the potentiometric experimental data carried out on Mn^2+^-*Amox* solutions has provided the results listed in Table 1. The speciation model obtained for Mn^2+^(M)-*Amox*(L) system, under all the investigated ionic strength and temperature conditions, includes only complex species with 1:1 M:L ratio, i.e., MLH_2_ and MLH. The speciation diagram regarding this system at C_M_ = 2 mmol L^−1^, C_L_ = 4 mmol L^−1^, *t* = 25 °C, *I* = 0.15 mol L^−1^ in NaCl is depicted in Figure 5. It is observed that MLH_2_ species predominates in the acid pH range, 3 ≤ pH ≤ 6.5, with metal fractions close to 0.6. MLH is the main species in the range 7 ≤ pH ≤ 9, with a maximum metal fraction almost equal to 0.8 at pH = 8.5. M_2_(OH)_3_ hydrolytic species does not reach significant formation percentages up to pH = 9.

**Figure 6:**
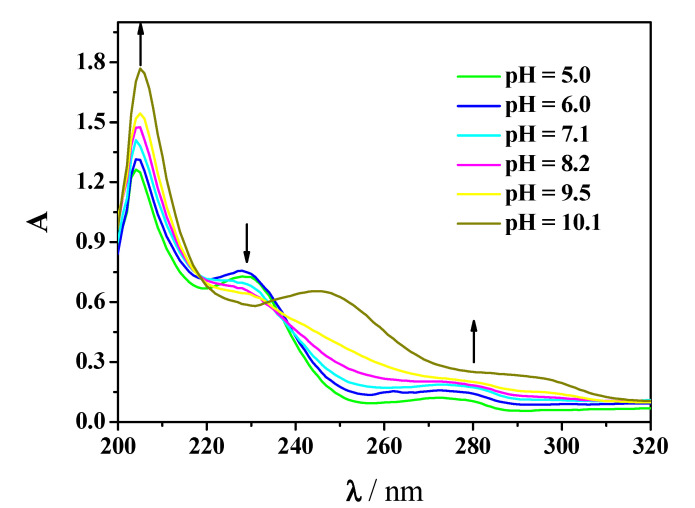
Experimental spectra at different pH on solutions containing Mn^2+^(M) and *Amox*(L) at C_M_ = 0.05 mmol L^−1^ and C_L_ = 0.075 mmol L^−1^, *t* = 25 °C, *I* = 0.15 mol L^−1^ in NaCl.

As for *Amp*, the system containing Mn^2+^-*Amox* has been investigated with potentiometry as well as with UV spectrophotometry. Experimental UV spectra at different pH values recorded on solutions containing Mn^2+^(M) and *Amox*(L) are shown in Figure 6 (C_M_ = 0.05 mmol L^−1^, C_L_ = 0.075 mmol L^−1^, *t* = 25 °C, *I* = 0.15 mol L^−1^ in NaCl). As known, spectra of solutions containing *Amox* result more complex than those containing *Amp*. In the range λ = 220–310 nm the former present intense bands, as expected for ligands having heteroatoms, linked to benzene ring, giving electronic transfer phenomena. Accordingly, the observed absorptions are typical of aromatic compounds with polar substituents, such as phenolic groups, characterized by charge transfer with ε = 5 × 10^3^–16 × 10^3^ L mol^−1^ cm^−1^ in the range mentioned above.

The UV spectra in terms of absorptivity, shape and band position are influenced by alkyl substitution, which generally causes a bathochromic shift of the phenolic band [35]. The presence of Mn^2+^ causes a slight hypsocromic shift of the phenolic band which can be explained by admitting metal-ligand interaction. More in detail, in the UV spectra shown in Figure 6 there are three maxima in the range of pH = 5–9.5, at λ = 204, 229 and 272 nm, at pH = 10.5 the maxima are at λ = 207, 248, 289 nm.

**Figure 7:**
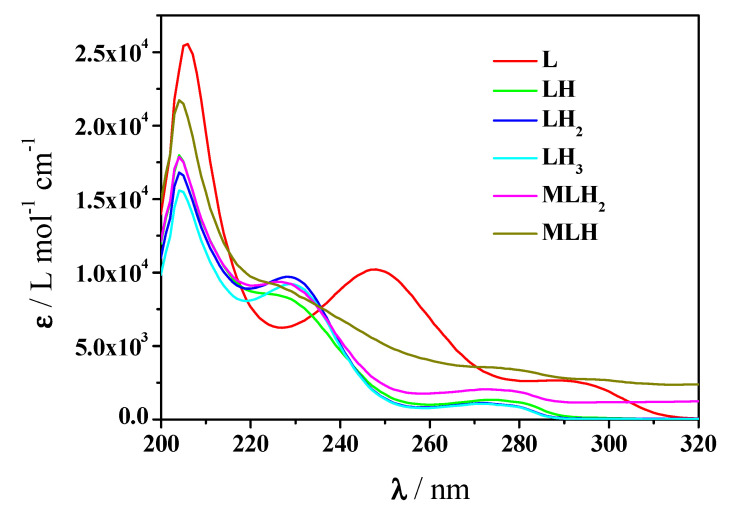
Molar absorbance values of Mn^2+^-*Amox* complexes and protonated and unprotonated *Amox* species at *t* = 25 °C and *I* = 0.15 mol L^−1^ in NaCl (charges omitted for simplicity).

These absorption maxima show different trends with increasing pH, the maxima at about 204 and 272 nm undergo a hyperchromic effect, on the contrary the maximum at 229 nm has a hypochromic effect. Molar absorbances of Mn^2+^-*Amox* species as well as those of *Amox* are depicted in Figure 7. More in detail, at λ = 207 nm, fully deprotonated ligand species has ε ≅ 27 × 10^3^ L mol^−1^ cm^−1^, followed by MLH and MLH_2_ with ε ≅ 21 × 10^3^, 18 × 10^3^ L mol^−1^ cm^−1^, respectively at λ = 204 nm. At λ = 229 nm, only ligand protonated species and complex ones present a maximum. LH_2_ species shows ε ≅ 1 × 10^3^ L mol^−1^ cm^−1^ at 229 nm, the interaction with metal causes a slight shift of the maximum. Accordingly, MLH_2_ and MLH present a maximum at 228 nm with ε ≅ 9 × 10^3^.

The results obtained by processing the spectrophotometric experimental data are listed in Table 2 together with the potentiometric ones under the same conditions of ionic strength and temperature. From the comparison, it is possible to affirm that spectrophotometry confirmed the same speciation model gained by potentiometry and the formation constants of both complex species were calculated with values quite similar to potentiometric ones.

### 2.2. Dependence of Formation Constants on Ionic Strength

The experimental results of Table 1, at various ionic strength values, were analyzed considering the Debye-Hückel equation:

(3) $$\log\beta ={\log\beta }^{0}-0.51z^{*}\frac{\sqrt{I}}{1+1.5\sqrt{I}}+CI$$

where β^0^ is the formation constant at infinite dilution, z* = ∑(charge)^2^_reactants_ − ∑(charge)^2^_products_, *C* is an empirical parameter. Several examples of these applications are reported in previous papers [36,37,38,39,40]. Calculated values of the formation constants at infinite dilution as well as of the *C* parameter for the dependence on the ionic strength are listed in Table 3. These parameters, necessary to calculate the stability constants at other ionic strengths, are of great importance for applications to real systems, such as biological fluids or natural waters. Recalculated values are listed in Table S3 of Supplemtary Materials.

By using the experimental results of Table 1, the speciation diagrams at *I* = 0.15 and 1 mol L^−1^ were obtained, as depicted in Figure 2 and Figure 5.

### 2.3. Dependence of Formation Constants on Temperature

Potentiometric titrations were performed at *t* = 15, 25, 37 °C and *I* = 0.15 mol L^−1^ for both Mn^2+^-*Amp* and Mn^2+^-*Amox* systems. The formation constant values at the various temperatures investigated are listed in Table 1, for both the systems. These data were analyzed using the van’t Hoff equation:

logβ_T_ = logβ_θ_ + Δ*H*^0^(1/θ − 1/*T*) Rln10 (4)

where logβ_T_ is the formation constant at a temperature *T* expressed in Kelvin, logβ_θ_ is the formation constant at *T* = 298.15 K, Δ*H*^0^ is expressed in kJ mol^−1^, R = 8.314472 J K^−1^ mol^−1^.

Enthalpy change values relating to all species of Mn^2+^-*Amp* and Mn^2+^-*Amox* systems were determined by using Equation (4). These values, together with those of free energy and entropy changes, are reported in Table 4, referring both on global (1) and on partial reaction (2). For Mn^2+^-*Amp* complexes Δ*H* values are exothermic for both the species. The trend of the thermodynamic parameters of Mn^2+^-*Amp* species is the same of Mg^2+^, the cation having the most similar biological behaviour to Mn^2+^, More in detail, for ML species of Mg^2+^-*Amp* system Δ*H* = −31.1 kJ mol^−1^, *T*Δ*S* = −12.6 kJ mol^−1^ (at *t* = 25 °C and *I* = 0 mol L^−1^), fairly similar to Δ*H* = −21 kJ mol^−1^, TΔ*S* = −8 kJ mol^−1^ here reported for the same species containing Mn^2+^.

**Figure 8:**
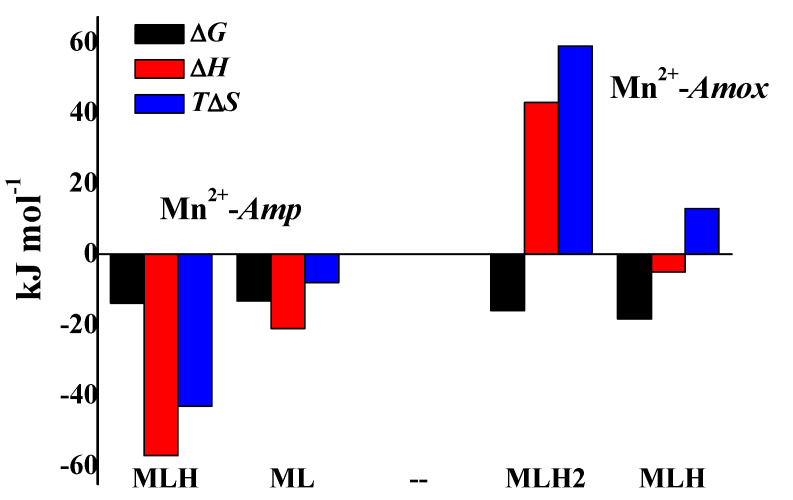
Bar plot of Δ*G,* Δ*H*, *T*Δ*S* referring to the Mn^2+^-*Amp* and Mn^2+^-*Amox* systems at *t* = 25 °C, *I* = 0.15 mol L^−1^ in NaCl, according to the reaction (2).

For Mn^2+^-*Amox* complexes, by considering partial reaction (2), Δ*H* value is exothermic for MLH species and endothermic for MLH_2_. Referring to partial reactions, the prevalence of the enthalpy and the entropic contribution for the species containing *Amp* and *Amox*, respectively, is not marked at all. Figure 8, represents a bar plot for both the systems which evidences the trend of thermodynamic parameters.

**Figure 9:**
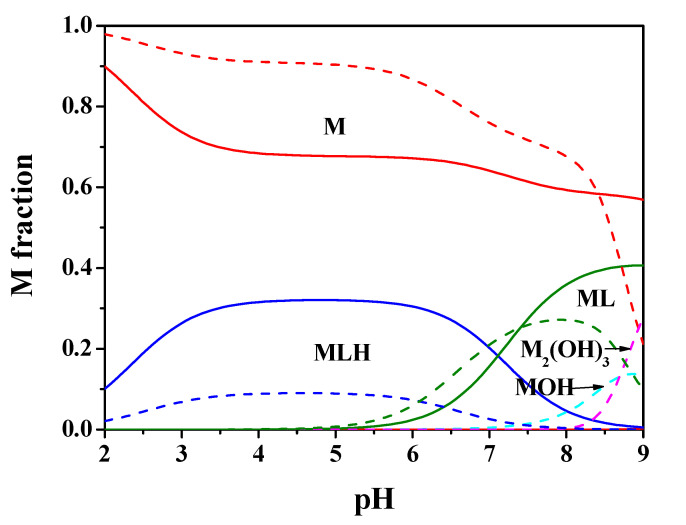
Speciation diagram of Mn^2+^-*Amp*(L) system, at C_M_ = 2 mmol L^−1^, C_L_ = 4 mmol L^−1^, *I* = 0.15 mol L^−1^, *t* = 15 °C (solid line), *t* = 37 °C (dashed line).

**Figure 10:**
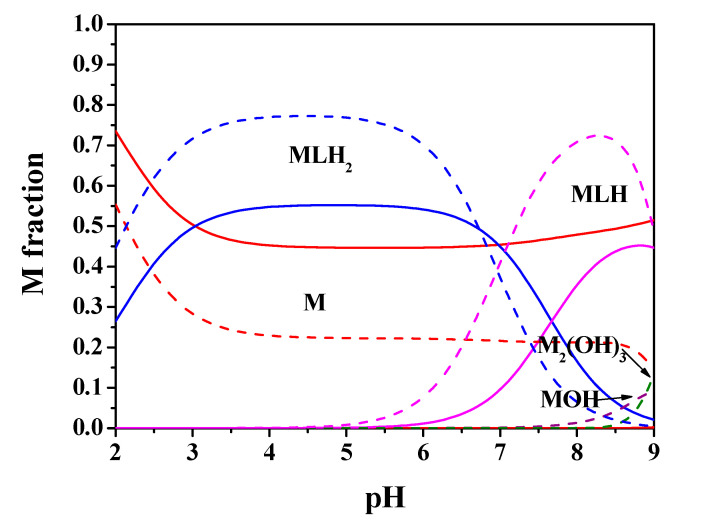
Speciation diagram of Mn^2+^-*Amox*(L) system, at C_M_ = 2 mmol L^−1^, C_L_ = 4 mmol L^−1^, *I* = 0.15 mol L^−1^, *t* = 15 °C (solid line), *t* = 37 °C (dashed line).

Figure 9 and Figure 10 show the speciation diagrams at *t* = 15, 37 °C relating to Mn^2+^-*Amp* and Mn^2+^-*Amox* systems, respectively. In both systems the change in temperature leads very different formation percentages of complex species, highlighting the need to take into account this parameter for a correct speciation study.

### 2.4. Sequestering Ability

**Figure 11:**
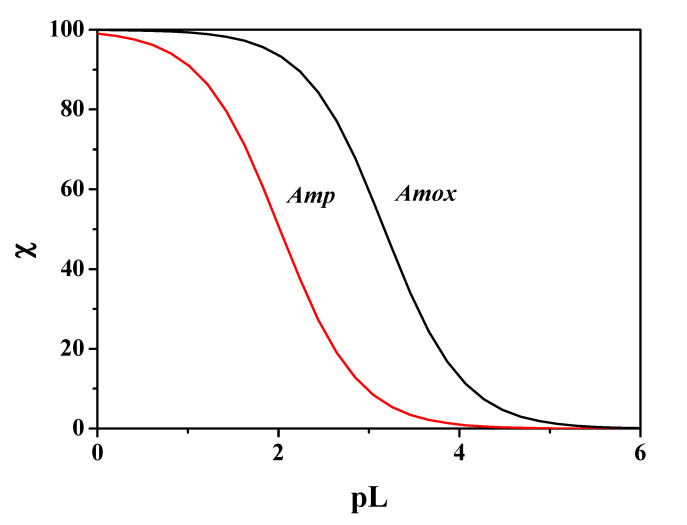
Sum of the fractions of Mn^2+^-*Amp* (in red), Mn^2+^-*Amox* (in black) species at pH = 7.4, *t* = 37 °C, *I* = 0.15 mol L^−1^ in NaCl.

Often the simple comparison of the formation constant values and the formation percentages of the species is not sufficient to analyze the higher or lesser sequestering capacity of a ligand towards different metal cations or of different ligands towards the same metal cation. In order to assess the sequestering ability, it is necessary to consider all the equilibria in which the ligands and various metal cations participate, such as hydrolysis reactions, ligand protonations, weak interactions of the ligand with the cations of the background salt. Several years ago, an empirical parameter, pL_0.5_, widely tested on multiple metal-ligand systems, was proposed for this purpose. pL_0.5_ represents the cologarithm of the concentration of the ligand able to bind 50% of the metal cation in traces, under the investigated conditions of pH, temperature and ionic strength. The following Boltzmann equation with asymptotes of 0 for pL→0 and 1 for pL→∞ is used to quantitatively define the sequestering capacity of a ligand against a specific metal cation [41,42,43,44,45]:

(5) $$\chi =\frac{1}{1+10^{\left(pL-pL_{0.5}\right)}}$$

where χ is the sum of the molar fractions of the species and pL represents the cologarithm of the total ligand concentration. In this way, the whole composition of the system is taken into account by pL_0.5_, as it depends on the system conditions (e.g., pH, temperature, ionic strength), considering all competitive reactions of the metal cation and the ligand. Moreover, in the calculation of the sequestering capacity a concentration of metal cation in traces was considered, making this parameter closer to the concentration conditions of many metal cations in real systems, such as body fluids. Under physiological conditions (pH = 7.4, *t* = 37 °C, *I* = 0.15 mol L^−1^), the sequestering ability values calculated against Mn^2+^ for *Amp* and *Amox* resulted in pL_0.5_ = 2.01, 3.17, respectively. It emerges that under physiological conditions the binding capacity of *Amox* is more than one logarithmic unit compared to *Amp*. These results are comparable with those obtained with another divalent cation, Ca^2+^, under the same conditions, pL_0.5_ = 1.82, 2.88 for *Amp* and *Amox*, respectively [22]. Another comparison can be made with Zn^2+^, in this case in the same conditions the sequestering ability is comparable for *Amox*, while for *Amp* it is significantly different. More in detail, pL_0.5_ = 3.16, 2.88 for *Amp* and *Amox*, respectively, towards Zn^2+^ [19]. The sequestering ability values of *Amp* and *Amox* towards Mn^2+^ were calculated under different conditions of pH, temperature and ionic strength. These values are listed in Table 5. Figure 11 shows the comparison of the sequestering capacity of the two ligands towards Mn^2+^ under physiological conditions. Binding ability of *Amox* is significantly higher than *Amp* under these conditions.

### 2.5. Literature Comparisons

In literature, in the main databases that report thermodynamic data [46,47,48], there are no formation constant values for Mn^2+^-*Amp* and Mn^2+^-*Amox* species. Only formation constant values for Cu^2+^, Ni^2+^, Zn^2+^, Cd^2+^, Co^2+^ are found. As far as we know, no thermodynamic data are reported on the complex species formed by *Amox* with any metal cation, apart from the results already published on Ca^2+^, Mg^2+^, Cu^2+^, Zn^2+^ cations [19,20,21,22]. Therefore, it is possible to compare simply the results here reported on Mn^2+^ with those obtained with other divalent cations. Among these cations, the behaviour of Mn^2+^ is quite similar to Mg^2+^ and Ca^2+^. More in detail, for the system containing *Amp*, formation constant values for MgLH and MgL species are 2.54, 2.86, respectively (at *I* = 0.15 mol L^−1^ and *t* = 25 °C) [21], for the same species containing Mn^2+^ are 2.47, 2.37, respectively, as reported here under the same conditions. For the system containing *Amox*, formation constant values for MLH and ML species with Ca^2+^ are 2.69, 2.59, respectively (at *I* = 0.15 mol L^−1^ and *t* = 25 °C) [22], for the same species with Mn^2+^ are 2.78, 3.20, respectively, as reported here under the same conditions.

## 3. Materials and Methods

### 3.1. Materials

The solutions containing Mn^2+^ were prepared by weighing and dissolution of the corresponding salt, manganese(II) chloride tetrahydrate (Reagent Plus ≥ 99%, Sigma, Darmstadt, Germany) These solutions were standardized by titration with ethylenediamine tetraacetic acid disodium salt (EDTA) standard solution. Solutions containing ligand were obtained by weighing and dissolution of ampicillin anhydrous and amoxicillin trihydrate (analytical standard, Sigma-Aldrich), used without further purification. Ligand purity, checked by alkalimetric titration, resulted > 99%. Fresh solutions of ligands were prepared every day. Hydrochloric acid and sodium hydroxide solutions were prepared by dilution of the respective ampoules (Fluka, Munich, Germany) and after they were standardized with sodium carbonate (≥99.5%, Sigma-Aldrich) and potassium biphthalate (≥99.5%, Sigma-Aldrich), respectively, previously dried in an oven at 110 °C. Sodium hydroxide solutions were prepared very frequently and were stored in bottles containing soda lime traps. Sodium chloride solutions were obtained by weighing the salt (Sigma-Aldrich^®^, puriss.), previously dried at 110 °C. All the solutions were prepared using bidistilled water (conductivity < 0.1 μS cm^−1^) and grade A glassware.

### 3.2. Potentiometric Apparatus and Procedure

Potentiometric titrations were performed by two distinct systems including a Metrohm (Herisau, Switzerland)) model 809 Titrando potentiometer, a Metrohm LL-Unitrode WOC combined glass electrode, and an automatic dispenser Metrohm Dosino 800. The system just described was connected to a PC and the measurements were acquired by the Metrohm TIAMO 2.2 software, which can control titrant delivery, e.m.f. stability, and data acquisition. Estimated accuracy of this system is ±0.15 mV and ±0.002 mL for e.m.f. and for titrant volume readings, respectively.

During the titration NaOH standard was added to 25 mL of the solution containing Mn^2+^, ligand and a supporting electrolyte (NaCl). For Mn^2+^(M)-*Amp*(L) solutions, 1 ≤ C_M_/mmol L^−1^ ≤ 3, 1 ≤ C_L_/mmol L^−1^ ≤ 6 and concentration ratios 1 ≤ C_L_/C_M_ ≤ 3. For Mn^2+^(M)-*Amox*(L) solutions, 1.5 ≤ C_M_/mmol L^−1^ ≤ 2, 2 ≤ C_L_/mmol L^−1^ ≤ 4 and concentration ratios 1 ≤ C_L_/C_M_ ≤ 2. The investigated pH ranges between 2 and 10. The measurements were performed into glass jacket thermostated cells, under different conditions of temperature (15 ≤ *t*/°C ≤ 37) and ionic strength (0.15 ≤ *I*/mol L^−1^ ≤ 1.0), under magnetic stirring and by bubbling pure N_2_ in order to avoid CO_2_ and O_2_ inside the solutions. An independent titration of HCl with standard NaOH was carried out for each measurement to calculate the pK_w_ value and the standard electrode potential E^0^ under the same experimental conditions of ionic strength and temperature of the measurement itself.

### 3.3. UV-Vis Apparatus and Procedure

A Varian (Agilent Scientific Instruments (Santa Clara, CA, USA)) Cary 50 UV-Vis spectrophotometer equipped with an optical fiber probe having a path length equal to 1 cm, was employed for spectrophotometric titrations on Mn^2+^-*Amp* and Mn^2+^-*Amox* solutions. The instrument was connected to a PC for the acquisition of the UV spectra in the range from 200 to 320 nm. A volume of 25 mL of the solution containing the metal cation and the ligand at different metal/ligand ratios was investigated at *t* = 25 °C, *I* = 0.15 mol L^−1^ in NaCl and at different pH values. For Mn^2+^(M)-*Amp*(L) solutions, 0.04 ≤ C_M_/mmol L^−1^ ≤ 0.05, 0.05 ≤ C_L_/mmol L^−1^ ≤ 0.08 and concentration ratios 1 ≤ C_L_/C_M_ ≤ 2. For Mn^2+^(M)-*Amox*(L) solutions, 0.05 ≤ C_M_/mmol L^−1^ ≤ 0.1, 0.075 ≤ C_L_/mmol L^−1^ ≤ 0.12 and concentration ratios 1 ≤ C_L_/C_M_ ≤ 2. The titrations were performed by potentiometric apparatus already described in the 3.2 paragraph. Aliquots of standard NaOH were added to solution containing Mn^2+^-*Amp* and Mn^2+^-*Amox* in order to reach the maximum absorbance of each complex species.

### 3.4. Calculations

Experimental potentiometric data were processed via the BSTAC and STACO programs, in order to gain the best speciation model for the systems under study, the formation constant values of the complex species, and all the parameters of an acid-base titration (analytical concentration of the reagents, standard potential E^0^, junction potential). LIANA software was employed in order to obtain the parameters for the dependence of complex formation constants on temperature and ionic strength. Details on software used in the refinement of the experimental data are reported in ref. [49]. Experimental spectrophotometric data were refined by HypSpec program to calculate the molar absorbance spectrum and the stability constant values of complex species [50,51]. HySS program was employed to obtain the speciation diagrams and the formation percentages of the complex species [52].

## 4. Conclusions

Due to the widespread use of aminopenicillins as antibiotics, knowledge of their complexation behavior and speciation is of fundamental importance for understanding their pharmacokinetics and pharmacodynamics. Speciation studies on Mn^2+^-*Amp* and *Amox* solutions were performed by potentiometry under different experimental conditions of temperature and ionic strengths. The results in terms of most reliable speciation model and formation constant values were fully confirmed by UV spectrophotometry for both the systems. More in detail, despite the different metal and ligand concentrations employed with these two techniques, a satisfactory agreement among the stability constants was achieved. The dependence of the stability constants on temperature and on ionic strength was studied. Accordingly, enthalpy changes at *t* = 25 °C and parameters for the dependence on ionic strength were also calculated.

For the correct assessment of the bioavailability of a drug or a trace element in the presence of that drug, it is essential to know the possible interactions occurring in body fluids. Accordingly, the knowledge of speciation and sequestering ability of two drugs employed as antibiotics, such as *Amp* and *Amox* with Mn^2+^, are necessary to evaluate the action mechanism of these ligands and the bioavailability of the metal cation in the presence of the drugs under physiological conditions. The assessment of the sequestering capacity is of fundamental importance as it takes into account the competitive processes of the metal cation and the ligand and facilitates comparisons among several ligands towards the same metal cation or the same ligand towards several cations under the same conditions of pH, temperature and ionic strength. The sequestering ability of *Amp* and *Amox* towards Mn^2+^ was evaluated under different conditions, including physiological ones. Under these conditions *Amox* has shown a higher binding capacity towards Mn^2+^ of more than one logarithmic unit compared to *Amp*.

## Figures and Tables

**Table 1:** Experimental formation constant values for Mn^2+^-*Amp*(L), Mn^2+^-*Amox*(L) species obtained by potentiometry at different temperatures and ionic strengths.

L	Species			logβ ^a^		
		*t* = 15 °C		*t* = 25 °C		*t* = 37 °C
		*I* = 0.15 ^b^	*I* = 0.15 ^b^	*I* = 0.48 ^b^	*I* = 0.96 ^b^	*I* = 0.15 ^b^
*Amp*	MnLH	9.47(2) ^c^	9.47(1) ^c^	9.57(3) ^c^	8.68(8) ^c^	8.18(8) ^c^
	MnL	2.36(3)	2.32(2)	2.37(5)	2.14(3)	2.09(3)
*Amox*	MnLH_2_	19.92(2)	19.64(2)	19.48(4)	20.14(2)	19.55(2)
	MnLH	12.25(3)	12.76(1)	12.80(2)	12.44(4)	12.59(2)
				**log*K*^d^**		
*Amp*	MnLH	2.10	2.42	2.47	1.44	1.42
	MnL	2.36	2.32	2.37	2.14	2.09
*Amox*	MnLH_2_	2.63	2.78	2.67	3.17	3.16
	MnLH	2.54	3.20	3.32	2.91	3.19

^a^ Refer to the reaction (1); ^b^ in mol L^−1^; ^c^ ≥95% of confidence interval; ^d^ refer to the reaction (2).

**Table 2:** Comparison between experimental formation constant values of Mn^2+^-*Amp* species obtained by spectrophotometry and potentiometry at *t* = 25 °C and *I* = 0.15 mol L^−1^.

L	Species	logβ ^a^	
		Spectrophotometry	Potentiometry
*Amp*	MLH	9.53(2) ^b^	9.47(2) ^b^
	ML	2.30(2)	2.32(2)
*Amox*	MLH_2_	19.77(7)	19.64(2)
	MLH	12.96(3)	12.76(1)

^a^ Refer to the reaction (1); ^b^ ≥95% of confidence interval.

**Table 3:** Thermodynamic formation parameters for Mn^2+^-*Amp* and Mn^2+^-*Amox* species.

Species	L	logβ^0^ ^a^	*C* ^b^
MLH	*Amp*	10.0(1) ^c^	−0.8(2) ^c^
ML		2.88(4)	0.10(9)
MLH_2_	*Amox*	20.13(8)	1.2(1)
MLH		13.8(1)	0.4(2)

^a^ Refers to reaction (1); ^b^ Equation (3); ^c^ ≥95% of confidence interval.

**Table 4:** Thermodynamic formation parameters for Mn^2+^-*Amp*, Mn^2+^-*Amox* species at *t* = 25 °C and *I* = 0.15 mol L^−1^.

Species	L	−Δ*G* ^a,b^	Δ*H* ^a,b^	*T*Δ*S*^a,b^
MLH	*Amp*	54.1	−101(13) ^c^	−47
ML		13.2	−21(3)	−8
MLH_2_	*Amox*	112.1	−28(6)	84
MLH		72.8	20(5)	93
**Reaction**	**L**	**−Δ*G*^b^**	**Δ*H*^b^**	***T*Δ*S*^b^**
M + LH	*Amp*	13.8	−57	−43
M + L		13.2	−21	−8
M + LH_2_	*Amox*	15.9	43	59
M + LH		18.3	−5	13

^a^ Refer to the reaction (1); ^b^ Expressed in kJ mol^−1^; ^c^ ≥95% of confidence interval.

**Table 5:** pL_0.5_ values of *Amp* and *Amox* towards Mn^2+^ at different pH, ionic strengths and temperatures.

L	*t*/°C	*I*/mol L^−1^	pH	pL_0.5_
*Amp*	25	0.15	7.4	2.35
	25	0.5	7.4	2.41
	25	1	7.4	2.17
	15	0.15	7.4	2.28
	37	0.15	7.4	2.01
	37	0.15	8.0	2.04
	37	0.15	6.5	1.78
*Amox*	25	0.15	7.4	3.05
	25	0.5	7.4	3.13
	25	1	7.4	3.06
	15	0.15	7.4	2.60
	37	0.15	7.4	3.17
	37	0.15	8.0	3.14
	37	0.15	6.5	3.16

## Supplementary Materials

The following are available online, Table S1: Hydrolysis constant values of Mn^2+^ at different temperatures and ionic strengths in NaCl, Table S2: Protonation constant values of *Amp* and *Amox* at different temperatures and ionic strengths in NaCl, Table S3: Recalculated formation constant values for Mn^2+^-*Amp* and Mn^2+^-*Amox* species at different ionic strengths and temperatures.
