# Supplementary material for: Interaction of Ampicillin and Amoxicillin with Mn2+: A Speciation Study in Aqueous Solution
Source: Molecules. 2020 Jul 8;25(14):3110. doi: 10.3390/molecules25143110 (PMC7397012; doi:10.3390/molecules25143110)
Supplement: Supplementary file 1 [file molecules-25-03110-s001.pdf]

Supplementary Material

Table S1. Hydrolysis constant values of Mn^{2+} at different temperatures and ionic strengths in NaCl.

Species	logβ [a)]				
	t = 15°C	*t* = 25°C			*t* = 37°C
	I = 0.15 [b)]	*I* = 0.15 [b)]	*I* = 0.48 [b)]	*I* = 0.96 [b)]	*I* = 0.15 [b)]
MOH^+	-10.6(1)	-10.46	-10.70	-10.95	-9.20(9)
$M_2(OH)_3^+$	-26.35(2)	-24.47	-24.69	-24.92	-23.51(4)
Ref.	c)	d)	d)	d)	c)

[a)] Refer to the reaction: $pM^{2+} + qH_2O = M_p(OH)_q^{(2p-q)} + qH^+$ [b)] in mol L^{-1}; [c)] unpublished data, this work; [d)] literature data.

Table S2. Protonation constant values of *Amp* and *Amox* at different temperatures and ionic strengths in NaCl.

			$\log\beta^{H}$			Ref.
t/°C	L	Species [a)]	I = 0.15 [b)]	I = 0.5 [b)]	I = 1 [b)]	
15	*Amp*	LH	7.317			[c)]
15		LH_2	9.899			
25		LH	7.051	7.095	7.244	
25		LH_2	9.638	9.660	9.805	
37		LH	6.757			
37		LH_2	9.351			
15	*Amox*	LH	9.706			[c)]
15		LH_2	17.287			
15		LH_3	19.783			
25		LH	9.565	9.482	9.533	
25		LH_2	16.865	16.807	16.972	
25		LH_3	19.427	19.404	19.615	
37		LH	9.400			
37		LH_2	16.395			
37		LH_3	19.049			

[a)] Refer to the reaction L + rH = LH_r, charges omitted for simplicity; [b)] in mol L^{-1}; [c)] F. Crea, D. Cucinotta, C. De Stefano, D. Milea, S. Sammartano, G. Vianelli Modelling solubility, acid–base properties and activity coefficients of 3 amoxicillin, ampicillin and (+)6-aminopenicillanic acid, in NaCl(aq) at different ionic strengths and temperatures Eur. J. Pharm. Sci. 47 (2012) 661-677.

Table S3. Recalculated formation constant values for Mn^{2+}-*Amp* and Mn^{2+}-*Amox* species at different ionic strengths and temperatures.

L	Species	logβ [a)]				
		t = 15°C	*t* = 25°C			*t* = 37°C
		I = 0.15 [b)]	*I* = 0.15 [b)]	*I* = 0.48 [b)]	*I* = 0.96 [b)]	*I* = 0.15 [b)]
Amp	MnLH	9.58(12) [c)]	9.52(14) [c)]	9.16(9) [c)]	8.73(13) [c)]	8.28(10) [c)]
	MnL	2.32(3)	2.23(9)	2.14(6)	2.18(9)	2.06(3)
Amox	$MnLH_2$	19.88(2)	19.63(5)	19.68(4)	20.11(4)	19.52(4)
	MnLH	12.43(5)	12.70(6)	12.59(6)	12.56(9)	12.68(4)

[a)] Refer to the reaction M+ L + rH = MLH_r, charges omitted for simplicity; [b)] in mol L^{-1}; [c)] ≥95% of confidence interval.
